# A method to mitigate spatio‐temporally varying task‐correlated motion artifacts from overt‐speech fMRI paradigms in aphasia

**DOI:** 10.1002/hbm.25280

**Published:** 2020-11-19

**Authors:** Venkatagiri Krishnamurthy, Lisa C. Krishnamurthy, M. Lawson Meadows, Mary K. Gale, Bing Ji, Kaundinya Gopinath, Bruce Crosson

**Affiliations:** ^1^ Center for Visual and Neurocognitive Rehabilitation Atlanta VAMC Decatur Georgia USA; ^2^ Department of Medicine, Division of Geriatrics and Gerontology Emory University Atlanta Georgia USA; ^3^ Department of Neurology Emory University Atlanta Georgia USA; ^4^ Department of Physics & Astronomy Georgia State University Atlanta Georgia USA; ^5^ Department of Biomedical Engineering Georgia Institute of Technology Atlanta Georgia USA; ^6^ Department of Radiology & Imaging Sciences Emory University Atlanta Georgia USA; ^7^ Department of Psychology Georgia State University Atlanta Georgia USA

**Keywords:** aphasia, ICA denoising, motion, motor, neglect, stroke, task fMRI

## Abstract

Quantifying accurate functional magnetic resonance imaging (fMRI) activation maps can be dampened by spatio‐temporally varying task‐correlated motion (TCM) artifacts in certain task paradigms (e.g., overt speech). Such real‐world tasks are relevant to characterize longitudinal brain reorganization poststroke, and removal of TCM artifacts is vital for improved clinical interpretation and translation. In this study, we developed a novel independent component analysis (ICA)‐based approach to denoise spatio‐temporally varying TCM artifacts in 14 persons with aphasia who participated in an overt language fMRI paradigm. We compared the new methodology with other existing approaches such as “standard” volume registration, nonselective motion correction ICA packages (i.e., AROMA), and combining the novel approach with AROMA. Results show that the proposed methodology outperforms other approaches in removing TCM‐related false positive activity (i.e., improved detectability power) with high spatial specificity. The proposed method was also effective in maintaining a balance between removal of TCM‐related trial‐by‐trial variability and signal retention. Finally, we show that the TCM artifact is related to clinical metrics, such as speech fluency and aphasia severity, and the implication of TCM denoising on such relationship is also discussed. Overall, our work suggests that routine bulkhead motion based denoising packages cannot effectively account for spatio‐temporally varying TCM. Further, the proposed TCM denoising approach requires a one‐time front‐end effort to hand label and train the classifiers that can be cost‐effectively utilized to denoise large clinical data sets.

## Graphical abstract

Quantifying accurate functional magnetic resonance imaging (fMRI) activation maps can be dampened by spatio‐temporally varying task‐correlated motion (TCM) artifacts in certain task paradigms (e.g., overt speech). In this study, we developed a novel independent component analysis (ICA)‐based approach to denoise spatio‐temporally varying TCM artifacts in fourteen persons with aphasia who participated in an overt language fMRI paradigm. Results show that the proposed methodology outperforms other approaches in removing TCM‐related false positive activity (i.e., improved detectability power) with high spatial specificity. The proposed method was also effective in maintaining a balance between removal of TCM‐related trial‐by‐trial variability and signal retention. Finally, we show that the TCM artifact is related to clinical metrics, such as speech fluency and aphasia severity, and the implication of TCM denoising on such relationship is also discussed.

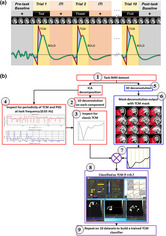

## 1 INTRODUCTION

Stroke is a devastating cerebrovascular disease that frequently results in motor, speech and language, and spatial neglect deficits. In order to understand stroke‐related neuroplasticity and monitor longitudinal brain changes in response to treatment, it is important to identify real‐world tasks that can be utilized within the magnetic resonance (MRI) that aides in characterizing stroke‐related impairment and recovery. For example, joystick‐based wrist movement paradigms in upper extremity motor stroke (Buetefisch, Revill, Shuster, Hines, & Parsons, 2014), attention modulation paradigms in right hemisphere stroke (Russell, Malhotra, Deidda, & Husain, 2013) and speech fluency‐based overt functional MRI (fMRI) in aphasia research (Benjamin et al., 2014) are clinically relevant task designs.

Head motion is always a concern in fMRI experiments, and indeed, task‐correlated head motion has been identified in motor fMRI paradigms (Kochiyama et al., 2005) and overt language fMRI paradigms (Gopinath et al., 2009; Xu et al., 2014). For overt‐language fMRI paradigms, the indirect source of task‐correlated motion (TCM) artifact stems from movement of the tongue, facial muscles, palate, and changes in air volume within the respiratory areas that can result in significant local magnetic field inhomogeneities (Birn, Bandettini, Cox, Jesmanowicz, & Shaker, 1998; Mehta, Grabowski, Razavi, Eaton, & Bolinger, 2006). Bulk head movement combined with speech‐related TCM can introduce complex spatio‐temporally varying motion artifacts that can be further exacerbated by through‐plane spin‐history effects and its interaction with susceptibility induced image distortions in fronto‐temporal brain areas (Kemeny, Ye, Birn, & Braun, 2005; Mehta et al., 2006). Another important note is that although these tasks are highly relevant to study stroke, TCM artifacts can be more exaggerated in stroke participants than the general population because of their neurological issues with movement of orofacial areas (such as in patients with oral apraxia and dysarthria), and relatively increased motion due to compensatory head and body movements while attending to overt language tasks. Thus, tasks such as overt‐language fMRI paradigms not only require correcting for bulk‐head motion but also nonrigid source of motion artifacts such as TCM.

Over the last two decades, there has been tremendous research effort to not only understand the nature of the spatio‐temporally varying TCM artifacts (Birn et al., 1998; Birn, Cox, & Bandettini, 2004) but also to develop approaches to mitigate such artifacts via improved data acquisition scheme (Bresch, Kim, Nayak, Byrd, & Narayanan, 2008; Narayanan, Nayak, Lee, Sethy, & Byrd, 2004), retrospective artifact removal methodologies (Birn et al., 2004; Birn, Bandettini, Cox, & Shaker, 1999; Bullmore et al., 1999; Gopinath et al., 2009; Xu et al., 2014), and improved design of experimental paradigms (Birn et al., 2004; Mehta et al., 2006). Signal changes due to speech can result in obscure false positive (FP) or Type I errors (Birn et al., 1999; Mehta et al., 2006) since both language‐related signal and speech‐related motion are synced with task stimuli and spatially co‐localize in frontal and temporal brain areas (Gopinath et al., 2009; Kemeny et al., 2005). Further, the large magnitude of speech‐induced TCM signal changes mask weaker true positive (TP) blood oxygen level‐dependent (BOLD) activity from key language areas (Gopinath et al., 2009). Establishing a cost (labor and computational)‐effective approach that can optimally minimize FP errors while retaining TP activity has been the challenging goal in this niche research area.

Independent component analysis (ICA) is a blind source separation technique in which the fMRI data are decomposed into spatial components with corresponding unique time courses that are maximally independent from each other. This attractive approach has been effectively developed for TCM removal by only few groups. (Kochiyama et al., 2005) focused on spatial ICA (s‐ICA) in young participants engaged in finger tapping task with and without head motion. More recently, Xu et al. (2014) developed a more sophisticated dual‐masked s‐ICA technique to denoise TCM in an overt‐language fMRI paradigm collected on healthy young participants. In the realm of routine bulkhead motion correction, Janssen and Mendieta (2020)) employed the standard ICA‐based denoising package (i.e., AROMA) to correct for motion in an overt picture naming task collected on healthy young subjects, while (Sebastian et al., 2016) utilized a rigid body alignment approach (i.e., MCFLIRT) to correct for motion in an overt picture naming task collected from acute stroke patients. However, given that speech related motion is not just spatially selective, but also temporally correlated with task‐induced hemodynamic response, and can vary from epoch to epoch (Gopinath et al., 2009), optimization of TCM denoising approaches to account for the noise jointly encoded in both spatial and temporal domains is equally important. Further, in neurological disease population such as stroke, these spatio‐temporally varying TCM artifacts can be exacerbated from patient to patient, and the number of lesion‐dependent ICs can be strongly dependent on lesion size (Yourganov, Fridriksson, Stark, & Rorden, 2018). Thus, the specific goals of this study are (a) to determine whether ICA‐based classifiers customized to capture spatio‐temporally varying speech‐induced TCM in patients with poststroke aphasia is effective in optimal denoising and (b) to determine the performance of such a classifier against other widely used motion correction approaches in aphasia research. We hypothesize that ICA classifiers trained to capture spatio‐temporally varying TCM in overt‐speech fMRI data sets acquired from persons with aphasia are more effective in mitigating the TCM artifacts as compared to other existing approaches, while also being cost efficient to reliably denoise larger data sets.

## 2 MATERIALS AND METHODS

### 2.1 Participants

Fourteen right‐handed participants (with English as their primary language) who were chronic stroke survivors (6 months or more post left‐hemisphere stroke) and diagnosed with aphasia (67 ± 11 years age, six females) were recruited into this study. For the purposes of the present report, only the baseline time‐point is presented. The demographic information of all 14 stroke participants is detailed in Table 1. Each participant was screened for MRI contraindications and provided written informed consent in accordance with procedures approved by the University of Florida Health Science Institutional Review Board. All consent procedures were in compliance with the Declaration of Helsinki. Subjects participated in an MRI session and a language assessment session which included administration of the Western Aphasia Battery ‐ Revised (WAB) (Kertesz, 2007). Table 1 also shows WAB fluency scores and aphasia quotient (AQ) which is an index of aphasia severity for all participants. Additional information such as lesion volume and a spatial overlap of lesion maps across all participants are provided in supplementary section S1.

**TABLE 1:** Demographic information of chronic stroke participants

ID	Age	Gender	CVA type	Lesion location	WAB AQ	WAB fluency	WAB classification
S01	79	Female	H	Subcortical	51.2	2	Broca's
S03	92	Female	I	Parietal	69.1	5	Conduction
S04	53	Male	I	Parietal, temporal	65.8	6	Conduction
S05	73	Male	I	Insula	76.4	5	Anomic
S06	62	Female	H	Subcortical	57.8	5	Conduction
S07	80	Female	I	Insula	78	6	Anomic
S08	55	Male	I	Frontal, parietal, subcortical, temporal	67.1	4	Transcortical motor
S10	69	Male	I	Frontal, parietal, subcortical	52.2	4	Broca's
S11	68	Male	I	Parietal, temporal	67.8	5	Conduction
S12	63	Female	I	Insula, temporal	66.6	6	Conduction
S14	61	Male	I	Subcortical	75.8	5	Anomic
S15	68	Female	I	Parietal	69.4	4	Broca's
S16	59	Male	H	Frontal, insula	74.5	5	Anomic
S19	64	Male	I	Insula	90	8	Anomic

*Note:* Reported are age, gender, cerebrovascular accident (CVA) type, lesion location, Western Aphasia Battery (WAB) aphasia qNouotient (AQ), WAB fluency, and WAB aphasia classification.

Abbreviations: H, hemorrhagic; I, ischemic.

### 2.2 MRI acquisition

The MRI data were acquired using a Philips 3 T Achieva scanner (Best, The Netherlands) using the body coil for radio frequency (RF) transmission and an 8‐channel head coil for RF receiving. The participant's head was comfortably stabilized using foam pads to minimize motion during and between scans. A 1 mm^3^ isotropic high resolution T1‐weighted anatomical image for spatial normalization to montreal neurological institute (MNI) template space was acquired using turbo field echo acquisition with the following parameters: echo time (TE) = 3.7 ms, repetition time (TR) = 8.1 ms, field of view (FOV) = 240 × 240 mm^2^, flip angle (FA) = 8°, and matrix size = 240 × 240. To identify areas of activation during overt word generation, six runs of continuous task fMRI time course was acquired with a BOLD weighted single shot gradient recalled echo planar imaging (EPI) sequence with the following parameters: 36 sagittal acquired slices, slice thickness = 4 mm, FOV = 240 × 240 mm^2^, matrix size = 64 × 64, TR = 1700 ms, TE = 30 ms, FA = 70°, acquisition bandwidth = 3,906 Hz/px, and 186 volumes per run.

### 2.3 Task design

**FIGURE 1:**
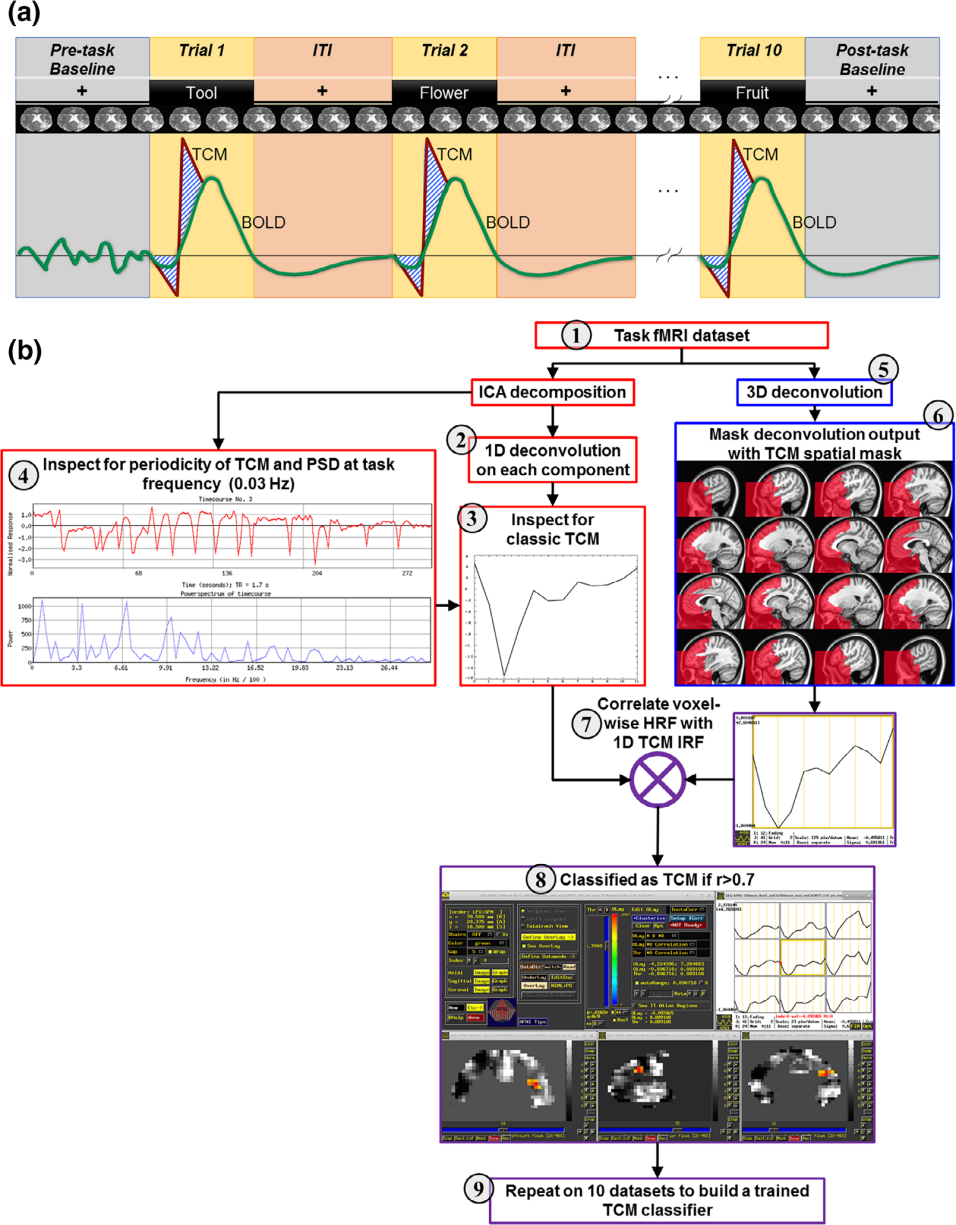
Description of the task, task‐correlated motion (TCM), and proposed methodology to correct for TCM. (a) Schematic of the overt language task functional magnetic resonance imaging (fMRI) design paired with task‐evoked hemodynamic response function (green) and TCM artifact (red). (b) Flowchart describing the novel spatio‐temporal independent component analysis (ICA)‐based TCM correction algorithm. The gray circle with numbers corresponds to bullet point description in the Section 2.4.2

To assess the patient's brain activity during word retrieval, fMRI data were collected during an overt category member generation task (i.e., semantic verbal fluency). Figure 1a shows the schematic for the task design. The task design comprised of six runs each containing 10 trials, for a total of 60 trials. The patients heard and silently read the category (e.g., “Tool”and were instructed to generate aloud a single exemplar (e.g., “Hammer”). The trial length was 6.8 s. During the inter‐trial intervals (ITI), patients viewed a fixation cross (“+”), and were instructed to not speak and stay still. The ITI was jittered between 13.6, 15.3, and 17 s. Also shown in Figure 1a is a schematic of the canonical BOLD signal (green line) along with the nuisance TCM artifact (red line) that is super‐imposed on top of the BOLD signal. Note the rapid signal changes (i.e., “spiky” signal change) due to TCM in comparison to the sluggish signal change of BOLD. This striking difference in the temporal signature will be utilized in the detection of TCM as described below.

### 2.4 Image processing

#### 2.4.1 Structural and task‐fMRI pre‐processing

The high‐resolution T1w images are denoised using an ONLM filter (Coupe et al., 2008; Wiest‐Daessle, Prima, Coupe, Morrissey, & Barillot, 2008) to remove Rician noise from the magnitude images (Gudbjartsson & Patz, 1995). The denoised T1w images are then bias field corrected, followed by the estimation of an initial binary lesion mask in native space using LINDA (Pustina et al., 2016). To skull strip the images, a binary brain mask was generated using optiBET (Lutkenhoff et al., 2014), manually touched up using ITK Snap to remove meninges and areas of calcification (Yushkevich et al., 2006), and applied to the bias field corrected T1w image. Finally, chimera spatial normalization (i.e., the lesion's right hemisphere homolog tissue was stitched into the lesion) was carried out to obtain more accurate transformation to MNI template space (Nachev, Coulthard, Jager, Kennard, & Husain, 2008; Yourganov et al., 2018).

The BOLD EPI images were processed systematically using a combination of AFNI (Cox, 1996), FSL (Smith et al., 2004), and Matlab (Natick, MA) in‐house scripts. The first nine TRs were discarded to ensure that the magnetization was at equilibrium, followed by slice timing and bulk head motion of the remaining fMRI time series. The volume registered BOLD data sets were then processed for TCM denoising in four different ways: (a) bare volume registered correction without any specific TCM correction (*Standard*), (b) novel TCM classifier (*TCMcorr*, details of the methodology described below), (c) routine ICA denoising package (*AROMA*) (Pruim et al., 2015), and (d) TCM classifier followed by AROMA (*TCMcorr + AROMA*). The denoised images were co‐registered to the T1w images using FSL's “*epi_reg*” boundary‐based registration and then warped to MNI space using the T1w to MNI transformation warp images obtained via FSL's “*FLIRT*” and “*FNIRT*” tools. For each data set, we also generated a binarized cerebrospinal fluid (CSF) mask that comprised of the ventricles (segmented from T1w images using FSL tools) and the lesioned brain area obtained through LINDA, all in MNI space. In the process of spatial smoothing of the BOLD images (Gaussian kernel size = 6 mm), the CSF mask was used in conjunction to minimize CSF contamination from ventricles and brain lesions. The smoothed BOLD time course was scaled with respect to the initial baseline condition (7 TRs = 11.9 s) to obtain task‐induced relative % BOLD change, censored for head motion (>0.3 mm), followed by deconvolution (AFNI's 3dDeconvolve) of the combined 60 trials across the six runs. In order to account for low‐frequency scanner drifts, we employed the polynomial fitting option within AFNI's 3dDeconvolve command. The deconvolution also incorporated regressors for the different motion parameter estimates and its derivatives obtained via *3dvolreg* and *1d_tool.py* AFNI tools. Finally, the statistical parametric activation maps on each individual subject were generated at an *R* ^2^ threshold of 0.16 (FWE corrected *p* < .01, cluster size = 30).

#### 2.4.2 Methodology for the novel spatio‐temporal TCM denoising algorithm

Figure 1b delineates the flowchart for our novel ICA‐based spatio‐temporal TCM denoising algorithm. Here is a step‐by‐step description of the algorithm flow: (a) the volume registered BOLD time series for each run was fed into FSL MELODIC for ICA decomposition (Beckmann & Smith, 2004), more details on the MELODIC setup can be found in the supplementary section S2). (b) stimulus‐locked 1D deconvolution was carried on each MELODIC IC time‐series. (c) Each component's impulse response function (IRF) was then inspected for classic TCM artifact (Birn et al., 1999; Gopinath et al., 2009), and the ICs that showed classic TCM artifact were noted. If an IC had a mixture of TCM noise and signal, we leaned on the conservative side to retain that IC for two purposes: (a) retain potential signal of interest (in other words, to avoid instances of “*throwing the baby out with the bathwater*”) and (b) toward robust training of the classifier that incorporates a variety of signal and noise components. (d) In conjunction to deconvolution based TCM IRF, the ICs were also inspected for “spiky” task periodicity in the IC time‐series and for spurious increase in power at task frequency (see supplementary section S3 for task frequency calculation) by looking at the power spectral density (PSD) plots. In addition to primarily customizing the hand classification to TCM, the process also involved identifying and labeling noise components related to susceptibility, physiological noise, and hardware artifacts based on recommended procedures by Griffanti et al. (2017). For consistency purposes, the hand classification was carried out by a single trained and experienced expert (V.K.).^1^ (e) In parallel, stimulus‐locked 3D deconvolution on the BOLD images (using all six runs) was carried out to obtain voxel‐wise HRFs. (f) A spatially selective TCM mask based on previous literature (Gopinath et al., 2009; Kemeny et al., 2005; Xu et al., 2014) was developed on standard 1 mm isotropic MNI brain (using ITKSNAP) and back projected to each individual participant's native space (using FSL tools). (g) The TCM spatial mask was then applied to the voxel‐wise activation maps to identify voxels in TCM‐prone areas. Each voxel's HRF within this TCM area were correlated with the 1D IRFs labeled as TCM obtained from step‐2, and the resulting Pearson's product moment for each voxel within the TCM area was thresholded at 0.7. (h) The ICs that survived the threshold (*r* = 0.7) from the previous step were labeled as noise (high correlation indicates TCM‐like signal changes), and the process was repeated on 10^2^ different data sets from the current study (sampled across the six runs and different subjects and time points) (Salimi‐Khorshidi et al., 2014). (i) The hand classification from 10 different data sets were fed into FSL's FIX tool to build a classifier based on FSL inbuilt machine‐learning algorithms (Salimi‐Khorshidi et al., 2014).

#### 2.4.3 HRF modeling to evaluate efficacy of TCM correction

To quantitatively evaluate the efficacy of TCM correction for motion‐related spikes in temporal dynamics, we first built a canonical HRF adapted from (Lindquist, Meng Loh, Atlas, & Wager, 2009) as shown below:

(1) $$h\left(t\right)=-A\left(\frac{t^{\alpha_{1}-1}\beta_{1}^{\alpha_{1}}e^{-\beta_{1}t}}{г\left(\alpha_{1}\right)}-c\frac{t^{\alpha_{2}-1}\beta_{2}^{\alpha_{1}}e^{-\beta_{2}t}}{г\left(\alpha_{2}\right)}\right)$$

where the canonical HRF is a linear combination of two different gamma functions (г), *A* controls the amplitude, *α* and *β* control the shape and scale of the HRF respectively, and *c* determines the ratio of the response to undershoot, with the following parameter values: *A* = 1, *α*_1_ = 2.5, *α*_2_ = 12, *β*_1_ = 0.4, *β*_2_ = 0.7, and *c* = 1/6.6. The estimated voxel‐wise HRF from the four different methodologies were then nonlinearly fitted to the canonical HRF using the generalized Levenberg–Marquardt nonlinear least squares algorithm as implemented in MATLAB (nlinfit), and goodness‐of‐fit estimate (*R* ^2^) was also quantified for each methodology.

#### 2.4.4 Coefficient of variation to compare denoising methodologies

In order to objectively compare the HRF temporal dynamics across all four methodologies, we identified significant surviving clusters (at an *R* ^2^ = 0.16) across all four methodologies from different subjects. We then drew a sphere (5 mm radius) centered at the peak activity and the average HRF was quantified from within that sphere. Within the same sphere, coefficient of variation (CoV) was computed on the smoothed time series (for each run and then average CoV across the runs) to quantitatively evaluate the effectiveness of denoising across the methodologies.

#### 2.4.5 Fano factor to characterize trial‐by‐trial variability

Fano Factor (FF) is a measure widely used in experimental cellular and molecular neuroscience fields to quantify the variability in spiking bursts (Falkner, Goldberg, & Krishna, 2013; Tolhurst, Movshon, & Thompson, 1981). Unlike CoV in which the mean and *SD* is calculated from the entire time series, FF involves mean (*μ*) and variance (*σ* ^*2*^) estimation from a defined time window (*W*) as shown as follows:

(2) $$\mathrm{FF}=\frac{\sigma_{W}^{2}\left(t\right)}{\mu_{W}\left(t\right)}$$

where *W* is defined as time length of first 3 TRs (=5.1 s) (Birn et al., 1998; Gopinath et al., 2009; Mehta et al., 2006) following the stimulus onset (see blue hatch in Figure 1a). For each subject, the FF was quantified from the smoothed BOLD time series (underneath the same spherical ROIs as described above) for each individual trial. The FF for each run was normalized and then averaged across the runs. Thus, a run‐averaged FF for each of the 10 trials is obtained, thereby allowing the quantitative investigation of continuous trial‐by‐trial variability during the first three TRs when the participant initiates speech.

#### 2.4.6 Quantification of denoising effects on spatial specificity and sensitivity

The task activation maps were thresholded at *R* ^2^ = 0.16 (cluster size = 30 voxels) and binarized to generate a mask of significant task activity for each analysis methodology (Standard, TCM, AROMA, and TCM + AROMA). The high resolution T1w images were segmented into white matter (WM) and gray matter (GM) using FSL's *FAST* tool and then were transformed into MNI space. Each task activation mask was then multiplied with the WM and GM masks to delineate significant task activity within WM and GM, respectively. The number of voxels that survived after this step were tabulated and compared across methodologies via *t*‐test using JMP Pro15 (SAS Inc., Cary, NC).

#### 2.4.7 Delineate the relationship between various clinical factors and TCM artifact

Table 1 outlines the values for clinical factors such as WAB AQ and WAB fluency, and supplementary section S1 outlines the lesion volume for each subject. The TCM artifact was quantified via FF using the Standard methodology. The assumption is that the Standard approach encodes more speech‐related TCM. Simple linear regression to assess the relationship between WAB fluency and TCM artifact was carried out in JMP Pro15 and reported with R^2^ and p values. Note that in order to derive a holistic perspective in the context of FF and CoV as described above, we will obtain these relationships from the same representative brain areas that were utilized in FF and CoV analyses. Further, we completed an ANOVA in JMP Pro15 to test whether aphasia severity (WAB AQ) and lesion volume explain TCM artifact. The results of the model are reported with F‐statistic and subsequent t‐tests are performed to determine which terms had significant effect.

## 3 RESULTS

### 3.1 Spatial and task specificity

**FIGURE 2:**
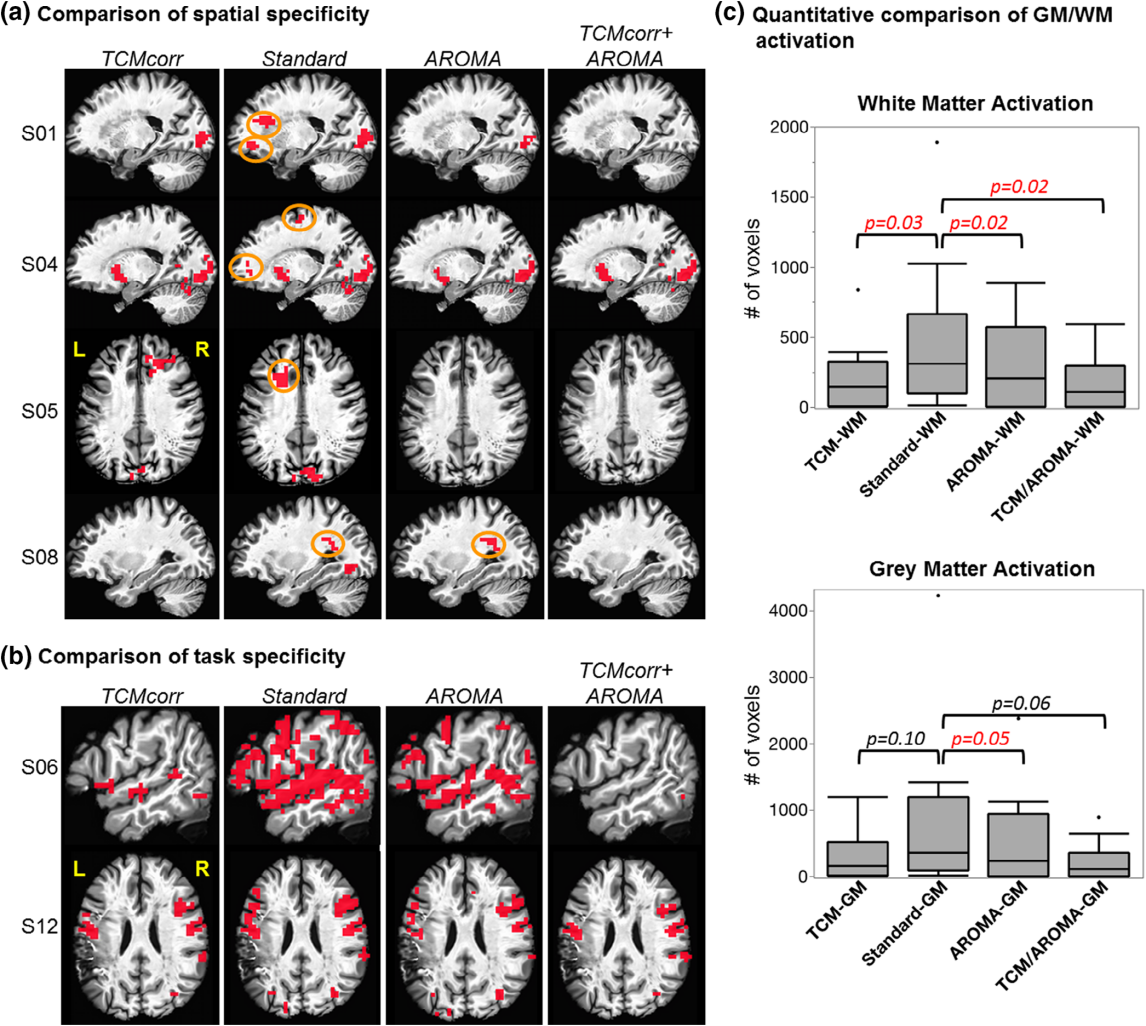
Comparison of spatial specificity across four different denoising methodologies. (a) Spatial specificity of each methodology to remove white matter activity. (b) Spatial specificity of each methodology to task‐relevant activation. (c) Quantification of retained gray matter and white matter voxels in thresholded (i.e., statistically significant) activation maps. The activation maps are significant at *R* ^2^ = 0.16, *p* < .01, cluster size = 30. Orange circles denote false positive white matter (WM) activity. L, left hemisphere; R, right hemisphere

Figure 2 demonstrates the performance of each methodology (TCMcorr, Standard, AROMA, and TCMcorr + AROMA) for spatial specificity across a sample of subjects with different lesion location and size. For the same statistical thresholding across the board (*R* ^2^ = 0.16, *p* < .01, cluster size = 30), Figure 2a shows spatial specificity to removal of unwanted white matter (WM) activity, and Figure 2b shows the task‐related spatial specificity. Visual inspection across the methodologies indicates that the novel methodology (TCMcorr) provides a good balance in removal of WM activity while maintaining task‐related spatial specificity. Figure 2c demonstrates group level quantitative comparison across methodologies of false positive white matter (WM) activity and gray matter (GM) activity in thresholded activation maps. Note that both TCMcorr and AROMA approaches remove false positive WM activity compared to Standard analysis routines, but TCMcorr does not significantly reduce the number of GM voxels, while AROMA does (Figure 2c). TCMcorr + AROMA removes excessive amount of signal along with noise. An axial montage of task activation focused around the lesion and language eloquent areas across all 14 subjects is demonstrated in supplementary section S7.

### 3.2 Task sensitivity

**FIGURE 3:**
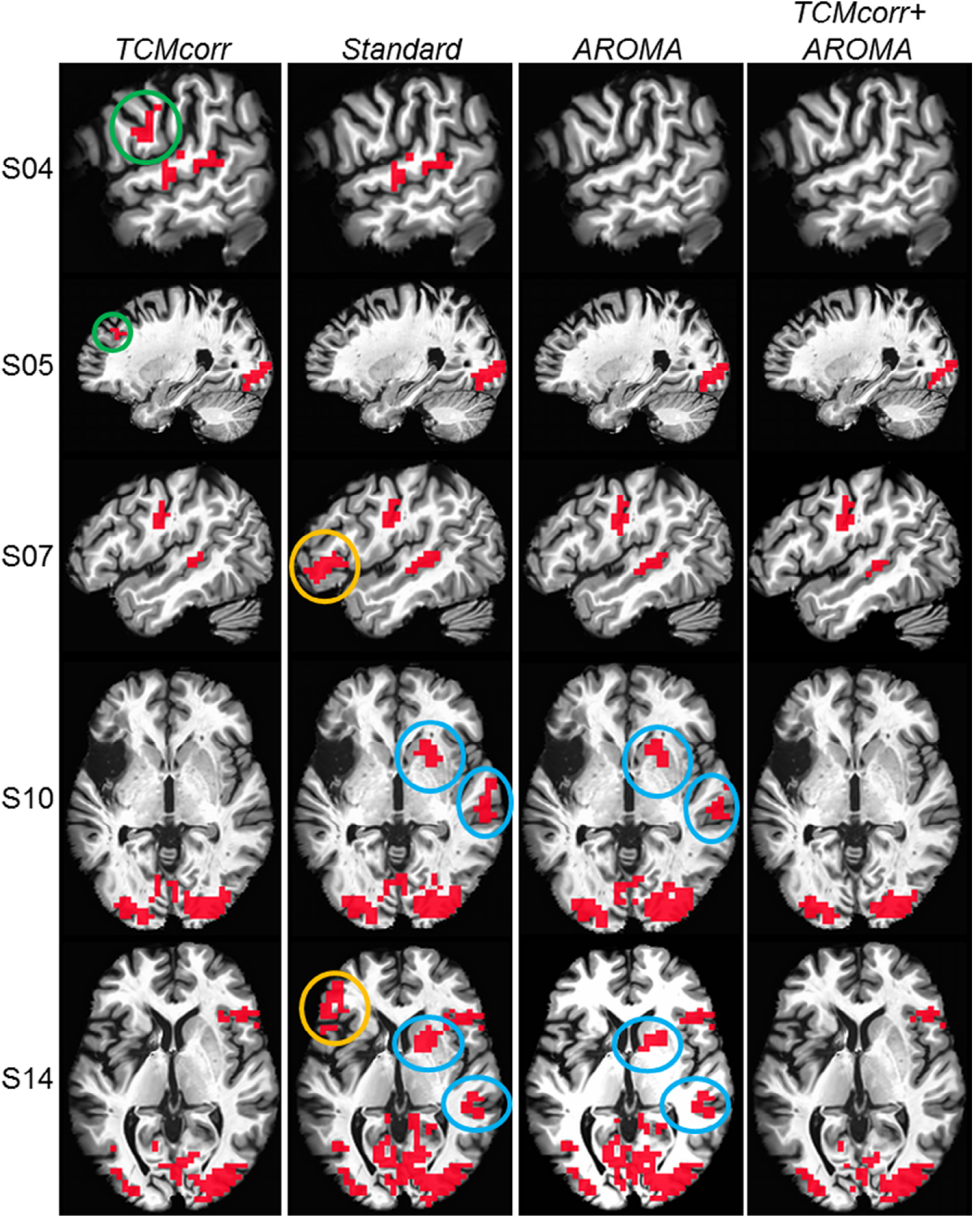
Comparison of task sensitivity across four different denoising methodologies. The activation maps are significant at *R* ^2^ = 0.16, *p* < .01, cluster size = 30. green circles denote true positive (TP) task‐relevant activation retained in the proposed novel methodology; orange circles denote potential false positive task‐correlated motion (TCM) activity, and blue circles denote TP activity retained in standard and AROMA methodologies. The MNI warped images are in the neurological convention

Figure 3 demonstrates the performance of each methodology for task sensitivity across a sample of subjects with different lesion location and size. The statistical thresholding (*R* ^2^ = 0.16, *p* < .01, cluster size = 30) was maintained the same across the board for fair comparison across the methodologies. The proposed TCM correction methodology was effective in bringing out task‐relevant true positive (TP) cortical activation in areas prone to TCM (see green circles), while the standard and AROMA methodologies were effective in retaining task‐relevant activation in subcortical and medial temporal areas (see blue circles) that are immune to TCM. The orange circles denote the FP TCM‐related activation that was observed in the standard approach. The combined TCMcorr + AROMA approach performed the worst where excessive task‐relevant signal was removed.

### 3.3 Effectiveness of the novel methodology in correcting for spatio‐temporally varying TCM

**FIGURE 4:**
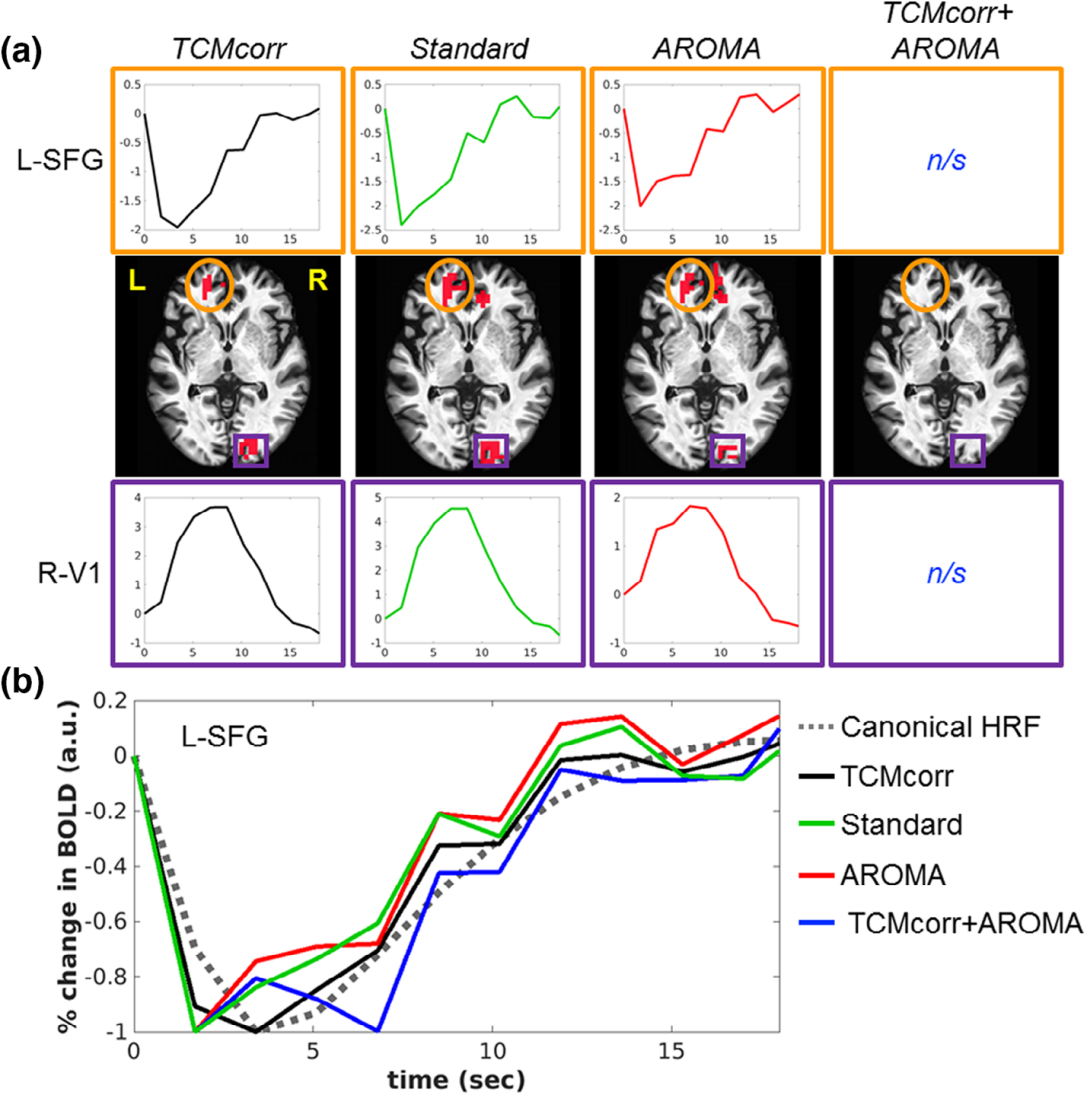
Demonstration of the effectiveness of proposed methodology to mitigate spatio‐temporal variability of task‐correlated motion (TCM) artifact in S01. (a) The activation and associated hemodynamic response function (HRF) from TCM prone area (L‐SFG, orange circle) and TCM immune area (R‐V1, purple box). (b) The comparison of the HRFs derived from each methodology to a canonical HRF. L, left hemisphere; L‐SFG, left superior frontal gyrus; R‐V1, right primary visual cortex; R,= right hemisphere; n/s, not significant at *R* ^2^ = 0.16, *p* < .01, cluster size = 30

Given that TCM is a spatio‐temporally varying artifact, it is relevant to dive deeper and more quantitatively explore the effectiveness of the proposed denoising approach, and how it compares to the other methodologies. Figure 4 shows the task activation map from a representative subject. The orange circle in Figure 4a denotes a TCM‐prone area (i.e., left superior frontal gyrus, L‐SFG), and its associated HRF across the methodologies. Figure 4b shows how the HRFs from the same area compared against the canonical HRF (dotted black line). Visual inspection of the HRFs shows that the proposed novel methodology effectively unmasks the BOLD‐like temporal dynamics while the other methodologies have TCM‐like “spiky” temporal artifacts. More quantitatively, the HRFs estimated across the methodologies were fitted to the canonical HRF and the goodness of fit (*R* ^2^) were as follows: *TCMcorr* (0.63), *Standard* (0.5), *AROMA* (0.27), and *TCMcorr + AROMA* (0.22) which is consistent with the qualitative inspection of the HRFs. A detailed summary of the *F*‐statistics and associated significance is shown on Supplementary section S4. Inspecting the modeled HRF parameters (see Supplementary section S3), specifically the ones that control for shape and scale (α and β), the proposed performance of the TCMcorr approach was by far superior compared to the other methodologies. Interestingly, activation from a TCM immune area (i.e., right primary visual cortex, R‐V1, shown in purple box) demonstrates BOLD‐like temporal dynamics across TCMcorr, Standard, and AROMA methodologies demonstrating that the TCM artifact is spatial selective and that the proposed methodology is superior in correcting the temporal dynamics in TCM prone areas, and leaving the BOLD temporal dynamics untouched (i.e., no over correction) in areas that are immune to TCM. The TCMcorr + AROMA methodology again performed the worst as it removed excessive amount of signal along with noise.

### 3.4 Comparison of HRFs derived from various TCM‐prone language‐relevant areas

**FIGURE 5:**
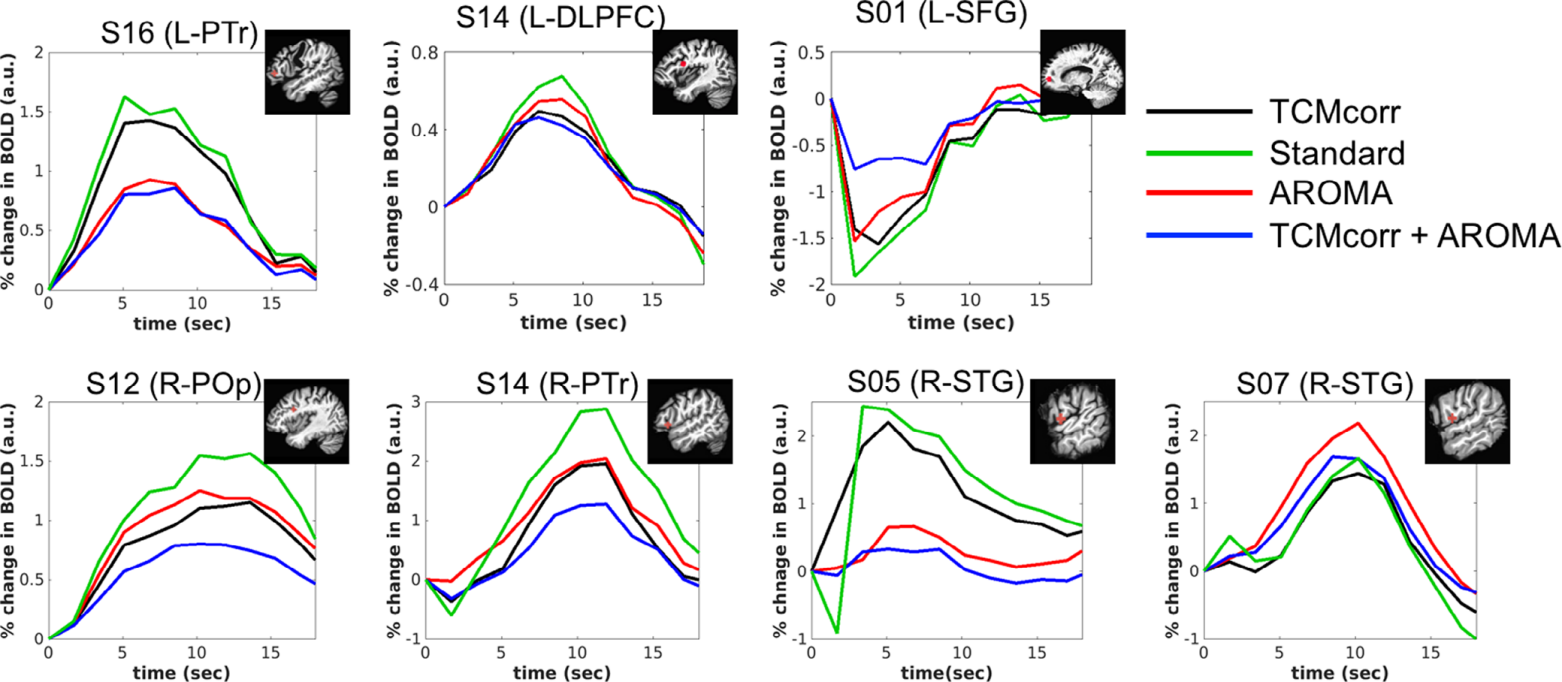
Comparison of hemodynamic response function (HRF) across four different denoising methodologies. Each HRF is derived from a task‐correlated motion (TCM) prone language area. DLPFC, dorsolateral prefrontal cortex; L, left; SFG, superior frontal gyrus; STG, superior temporal gyrus; POp, pars opercularis; PTr, pars triangularis; R, right. The naming of these specific regions of interest (ROI) was based on the center of peak activity for that ROI obtained via AFNI's cluster report

In the results above, note that TCMcorr + AROMA approach did not result in any significant activation at the set statistical threshold for S01. Figure 5 shows an objective comparison of HRF from TCM prone language areas that survived the statistical threshold across all four methodologies, for representative areas from both left and right hemisphere. Consistent with the results shown in Figure 4, the Standard approach shows TCM‐related temporal spiky artifacts (S05 and S14) while the novel TCMcorr approach corrects for those temporal artifacts. In terms of BOLD amplitude, we observe that TCMcorr + AROMA approach provides the least sensitivity followed by the AROMA approach, which may be due to excessive removal of signal along with noise. On the other hand, the standard approach has high signal to noise ratio (SNR) since it retains multiple sources of artifacts (such as motion, susceptibility‐motion, etc.) in addition to false positive TCM‐related activity.

### 3.5 Effectiveness of methodologies to account for trial‐by‐trial variability

**FIGURE 6:**
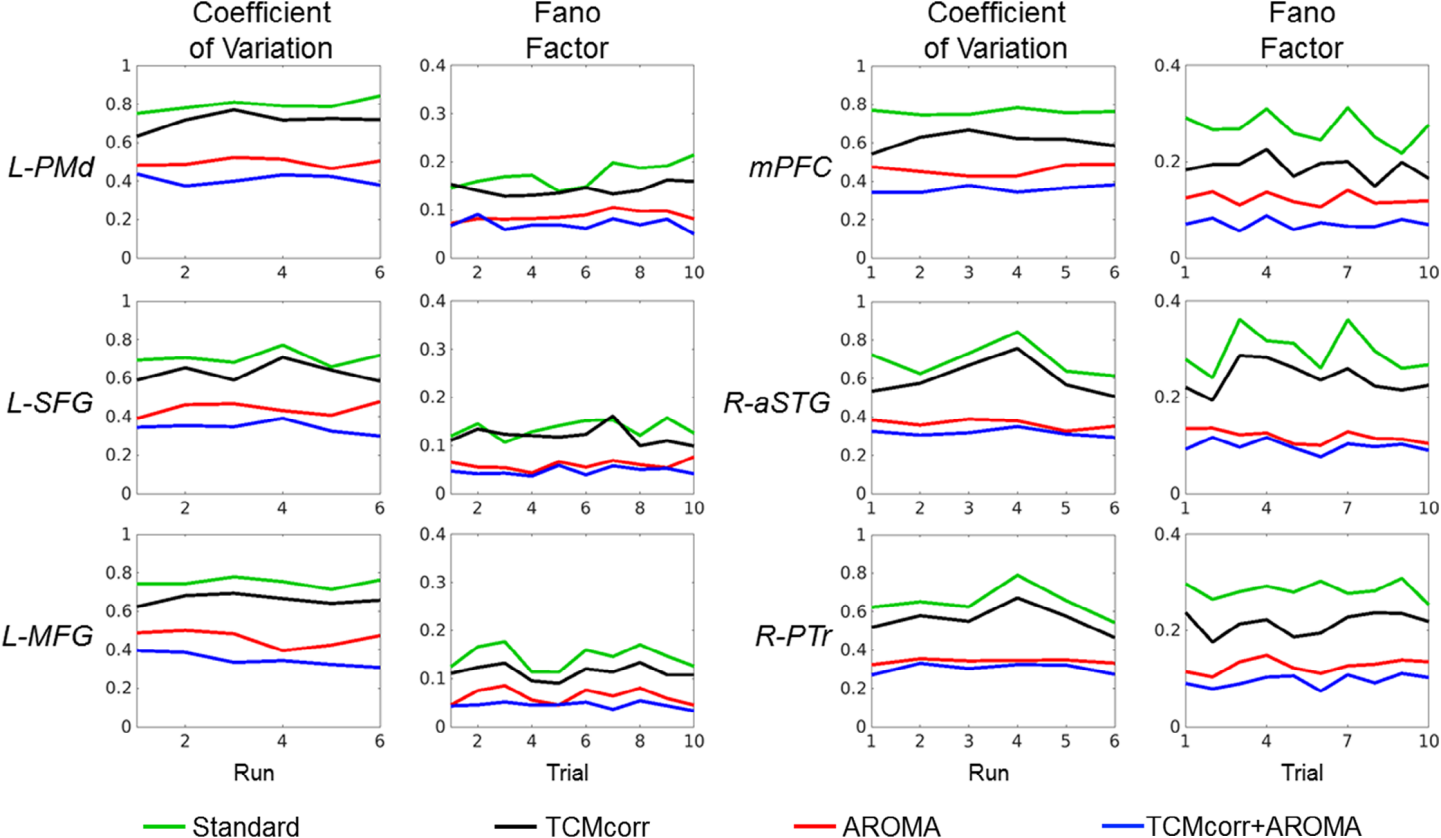
Comparison of trial‐by‐trial variability and CoV across four different denoising methodologies. For each of the six different TCM prone brain areas, the left column shows coefficient of variation (CoV) quantified across runs, and the right column depicts the trial‐by‐trial variability quantified using Fano Factor from the same brain areas. CoV, Coefficient of Variation; FF, Fano Factor; L, left; MFG, middle frontal gyrus; mPFC, medial pre‐frontal cortex; R, right; SFG, superior frontal gyrus; aSTG, right anterior superior frontal gyrus; PMd, dorsal pre‐motor cortex; PTr, pars triangularis

Average FF was quantified to represent signal variability at task‐onset from various representative TCM prone brain areas from intact left and right hemisphere areas (see Figure 6). A higher FF is associated with a greater amount of spikiness, that might be associated with TCM artifacts. As a function of trials (averaged across runs), the AROMA and TCMcorr + AROMA approaches demonstrate “flat” projections while the Standard approach shows more variations in FF. The flat projection suggests removal of speech‐induced TCM variability across the continuum of trials that are apparent in the Standard approach. Interestingly, the FF in intact left hemisphere areas is relatively lower compared to the right hemisphere areas. The proposed TCM correction approach (TCMcorr) has a FF that is reduced compared to Standard, representing removal of TCM but greater FF than AROMA and TCM + AROMA, representing retention of signal. In other words, TCMcorr potentially removes TCM variability while retaining the signal of interest. Another metric of quantifying signal variability is coefficient of variation (CoV) assessed at the run level (averaged over 10 trials). Similar to FF, the CoV also shows that TCMcorr provides a balance between removal of noise and retention of signal. Group level paired *t*‐test showed significant (*p* < .01) difference between the methodologies for both FF and CoV metrics.

## 4 DISCUSSION

TCM is a unique challenge that is inherent to certain types of task designs (e.g., overt speech) where there is shared variance between stimulus‐evoked task activity and noise. Treating TCM as rigid body motion artifacts and thereby approaches to correct for TCM using tools designed for routine motion artifacts, can be sub‐optimal at best, or misleading at worst for clinical research. Thus, in this study, we set out to develop ICA classifiers trained to capture spatio‐temporally varying TCM in an aphasia data set (with overt‐language fMRI paradigm) (Krishnamurthy, Krishnamurthy, Meadows, et al., 2020). We provided evidence that our novel TCMcorr methodology is more effective in mitigating TCM artifacts compared to other existing approaches, while also being cost efficient to reliably denoise larger data sets. From a clinical translation standpoint, optimal removal of TCM artifacts will allow for subsequent secondary analyses such as quantifying area under the curve (underneath the denoised HRF) to obtain longitudinal BOLD‐behavior relationships (Krishnamurthy, Krishnamurthy, Drucker, et al., 2020) indexing neuroplasticity in response to treatment and interventions.

Speech‐induced TCM is a spatio‐temporally varying artifact that shares variance with task‐induced neural activity. A previous report (Mehta et al., 2006) indicated that speech‐related residual variance increases by about 15% relative to covert (silent word generation) task, and that in some brain areas, these increases are much greater. Previous reports have also shown significant inter‐subject variability in spatial location of speech‐related variance (Barch et al., 1999; Preibisch et al., 2003) that includes frontal, inferior, and temporal brain areas (along with outside the brain) that progressively attenuate towards the posterior, superior, and medial regions including around the ventricles (Birn et al., 1999; Gopinath et al., 2009; Kemeny et al., 2005; Mehta et al., 2006; Xu et al., 2014). Thus, the spatio‐temporal variance in speech‐induced TCM severely affects the capability to detect true positive BOLD activity and separate it from false positive activity for brain areas relevant to language and cognitive processing. The inherent spatio‐temporal variance in TCM can be more exacerbated in patients with nonfluent aphasia as their speech execution (motor programming and execution) is damaged and can vary depending on lesion location and size. Although, there have been several elegant approaches in detecting and mitigating TCM (Birn et al., 1999; Bullmore et al., 1999; Gopinath et al., 2009; Kochiyama et al., 2005; Mehta et al., 2006; Xu et al., 2014), a cost‐effective (labor and computational) and automated approach to effectively denoise both spatial and temporal variance in TCM while maintaining high fidelity to task‐relevant signal that can be customized to variety of stroke data sets was not available until the current report.

ICA is a blind source separation technique that has been widely used in fMRI field for removal of various types of artifacts while preserving the integrity of continuous time‐series. (Du et al., 2016; Griffanti et al., 2017; Pruim et al., 2015; Salimi‐Khorshidi et al., 2014; Thomas, Harshman, & Menon, 2002). ICA‐based approaches exploiting both spatial and temporal information have been developed to identify and remove physiological noise (Beall & Lowe, 2007; Perlbarg et al., 2007), and rigid‐body head motion (Pruim et al., 2015; Yourganov et al., 2018). Although these techniques have been highly automated for easy user interface, these tools are optimized primarily for denoising physiological noise and rigid head motion from resting‐state fMRI data, but not TCM.

A handful of studies have utilized ICA to remove TCM, but were focused more on the spatial information of ICA‐decomposed fMRI data (Kochiyama et al., 2005; Xu et al., 2014). Our novel approach, on the other hand, accounts for both the spatial and temporal aspects of speech‐induced TCM. We utilized widely available FSL tools (MELODIC and FIX) (Beckmann & Smith, 2004; Salimi‐Khorshidi et al., 2014) as the basis for the ICA decomposition and classification, and further developed TCM‐specific denoising approach using an overt language task fMRI data set. In order to impose TCM‐specific spatial constraints, we developed a customizable approach wherein the TCM‐specific spatial mask was developed in MNI space that was back‐transformed to subject's native space for the masking of spatial independent components (s‐ICs). Note that for our mask, we not only include the classic TCM areas (i.e., frontal, inferior, and temporal areas) but also outside the brain as (Xu et al., 2014) have shown that expanding the mask to include extracerebral soft tissue and air cavities can aid in TCM‐relevant noise decomposition. On the IC time‐series, we ran stimulus‐locked deconvolution to obtain impulse response function (IRF) for each of those unique time‐series. The stimulus‐locked approach is meant to be simple (i.e., no need for complicated experimental setup for microphones to record and then to analyze the subjects' responses) for the purposes of TCM identification and denoising. In the process of ICA decomposition, we also did not impose variance normalization to maintain the detectability to TCM noise and signal of interest, and thereby their separability. In parallel, we also analyzed the 3D functional data for stimulus‐locked deconvolution, and the significant HRF underneath the TCM spatial mask (as described above) was correlated with each IC's IRF to ensure that the hand labeling of a given IC captured both the spatial and temporal aspects of TCM. In addition to spatial and temporal features, we also utilized the power spectral density plots (for each IC to identify spurious increase in power at task frequency) to facilitate the identification of TCM. Finally, to ensure that this novel approach is cost friendly (i.e., minimize the labor and computational resources to denoise each individual task run of N subjects across multiple time points in longitudinal studies), we utilized the ICA FIX tool to train a classifier that is then used to denoise the entire data set. To build a robust TCM‐classifier, it is important to use a minimum of 10 data sets (Salimi‐Khorshidi et al., 2014) and to select a wide variety of TCM artifacts such that when deployed on new data sets, the classifier can capture a broad spectrum of TCM noise. Such an approach will also be favorable to capture a wide variety of TCM artifacts that are influenced by neurological (in our case, language and speech) impediments in clinical datasets.

Given that AROMA is a widely used ICA‐based denoising approach, we wanted to dive deeper to compare how the novel TCMcorr approach compared to AROMA in mitigation of speech‐related TCM. Since AROMA was developed to correct for global bulkhead motion (Pruim et al., 2015), it is grossly non‐selective and thus was not able to remove speech‐related nonrigid motion artifacts (e.g., L‐SFG in Figure 5). It should be noted that the TCMcorr classifier was not only sensitized to TCM, but the hand classification also incorporated accounting for other sources of artifacts and thus when compared to AROMA, it provided either comparable or superior results in terms of artifact removal (e.g., removal of white matter artifacts in Figure 2). However, the task sensitivity in certain brain areas (subcortical and medial temporal) that inherently has lower SNR may have been compromised in the TCMcorr approach as compared to AROMA (Figure 3), and also the BOLD amplitude was higher for AROMA as compared to TCMcorr in some cortical areas (see R‐POp, and S07 R‐STG in Figure 5) in representative subjects, but the shape of the HRF is quite normal. On the other hand, in subjects with spiky HRFs (see L‐SFG and S05 R‐STG in Figure 5), the TCMcorr approach not only removes the spikiness but also retains higher BOLD magnitude compared to AROMA. Collectively, these results suggest that the novel TCMcorr approach is more tailored to effectively account for speech‐induced spatio‐temporally varying TCM. Also, inspecting the average CoV across all subjects (Figure 6), the TCMcorr approach provides a good balance between removal of TCM artifact while retaining signal of interest. Given that AROMA is tailored to bulkhead motion and TCMcorr sensitized to TCM, from a methodological standpoint, it was imperative to also combine the two approaches to see if that was more optimal than either alone. Across the board (see Figures 2, 3, 4, 5, 6), our results show that TCMcorr+AROMA approach removes excessive amount of structured signal along with structured noise resulting in “overcorrection” (Bright & Murphy, 2015; Krishnamurthy et al., 2018).

Although deconvolution is an elegant approach to quantify HRF, one down side to such an approach is that it combines all the trials for HRF estimation. In such an approach, deconvolution might be less sensitive to trial‐by‐trial variability in TCM. This issue is even more protracted in patients with aphasia as they have neurologic speech timing issues. It is also reasonable to expect filler word (e.g., ‐ “um, er”) or no response which may still involve stimulus‐locked cognitive processing and perhaps some degree of rigid and nonrigid movements. Thus, FF was quantified for each trial using the time‐series and window length of 3TRs (=5.1 s) (Birn et al., 1998; Gopinath et al., 2009; Mehta et al., 2006) synced with the stimulus onset. The FF quantified across all subjects from various language‐specific TCM prone areas show that the TCMcorr approach provides a good balance between signal detectability and minimizing TCM‐related trial‐by‐trial variability. Another interesting note is that the FF is relatively lower in left hemisphere residual areas as compared to right hemisphere intact language areas (see Figure 6). Given that FF is quantified from HRF (first three TRs) that was derived from stimulus‐locked deconvolution, lower FF in residual language areas from lesioned hemisphere might indicate lower and/or delayed activity. This could potentially be due to word initiation and word retrieval difficulties in these patients that need to be further investigated in future studies. Overall, it is promising to note that FF can be a potential biomarker to identify word initiation deficits (i.e., vascular lags; Siegel, Snyder, Ramsey, Shulman, & Corbetta, 2016) and how it may change as a function of language rehabilitation.

In light of clinical translatability of the proposed work, we also took a preliminary stab at exploring the relationship between TCM artifact (and its removal with proposed methodologies) with clinical factors (such as WAB fluency and WAB AQ) and lesion size. Note that the assumption here is that the FF derived from Standard approach has higher amount of TCM and thus served as the proxy for indexing TCM artifact. Results from supplementary section S5 indicate that lesion size does not predict speech‐induced TCM artifact. However, WAB fluency and WAB AQ (i.e., aphasia severity) did show a significant inverse relationship with TCM artifact. That is, increased aphasia severity and poorer performance on WAB speech fluency is predictive of a greater amount of TCM artifact in brain areas (i.e., R‐aSTG and L‐MFG) involved in fluency (Meinzer et al., 2009). In terms of how FF relates to clinical factors after the removal of TCM, we observe a spatial dependence across the methodologies. Although this is quite promising, given that our study had a small sample size, further work is required to investigate the validity and reliability (Wilson, Bautista, Yen, Lauderdale, & Eriksson, 2017) of overt task activation in patients with aphasia. Such an investigation should also incorporate delineating the impact of various analysis parameters such as voxel‐wise threshold and cluster size cutoff on test–retest reliability of such activations. In the current study, given that the focus was to compare the removal of TCM across methodologies, we chose a conservative approach to threshold at an R^2^ of 0.16 across the board. We also explored relative thresholding of *R* ^2^ in a few representative subjects (see supplementary section S6), and did not find any significant impact within the scope of the current study, but further work is warranted.

Overall, it is promising to note that ICA technique can be developed to account for both spatial and temporal variabilities that are unique to TCM induced by certain tasks. Such tasks include overt speech or joystick movement inside the scanner that are clinically relevant to stroke‐related neurorehabilitation, and thus cannot be easily pushed aside for resting‐state fMRI scans. Although fully automated approaches are attractive for clinical translation, careful identification of TCM‐related ICs require training and a one‐time front‐end effort from a researcher. The current work provides such a framework and is positioned towards developing and disseminating fully automated pipelines. From a validity standpoint, indeed further work is warranted to establish test–retest reliability on TCM denoising such that it increases confidence in deploying this approach for clinical use. From an acquisition standpoint, B0 field maps (Jezzard & Balaban, 1995) were unfortunately not acquired with this data set to correct for EPI geometric distortion. Although sagittal EPI acquisition was employed to minimize speech‐related TCM, most of the contemporary fMRI data collection involves multiband transverse acquisition that facilitates the use of “TOPUP” approach (Andersson, Skare, & Ashburner, 2003) to correct for EPI distortion. In the same context, although multiband acquisition can be attractive for capturing the temporal dynamics of TCM, multiband acquisition is inherently more sensitive to motion than traditional EPI scans. Given that an 8‐channel head coil was utilized, the sensitivity to deeper sub‐cortical areas was limited and may have affected optimal denoising in the proposed methodology. Thus, future work will entail development and optimization of the proposed methodology to effectively account for TCM in multiband fMRI data sets that utilizes contemporary 32‐channel receiving head coils. As a proof of principle, we have demonstrated that the proposed approach works on overt speech tasks, but the generalization of this approach requires further testing on other stroke rehabilitation relevant tasks such as joystick movement or spatial aiming tasks that can also have unique task‐specific TCM.

## 5 CONCLUSION

In summary, TCM is a unique spatio‐temporally varying artifact that can reduce the detectability of true positive activation. TCM artifacts cannot be mitigated by using routine denoising packages that are tailored to correct for rigid body bulkhead motion. We have demonstrated that developing a TCM‐specific ICA classifier that utilizes both spatial and temporal features can be spatially selective while also retaining and/or enhancing the detectability power to task‐induced BOLD activity. Importantly, our approach is optimized to account for trail‐by‐trial variability in TCM. Finally, the development of a TCM classifier is cost efficient to analyze large data sets designed to study longitudinal rehabilitation in different stroke populations.

## CONFLICT OF INTEREST

There is no conflict of interest among the authors and the author list is final.

## Supporting information


**Appendix** **S1.** Supporting Information.

## Data Availability

The data that support the findings of this study are available from the corresponding author upon reasonable request.

## Endnotes

^1^
The lead author (V.K.) hand classified the ICs based on his experience in identifying TCM from (Gopinath et al., 2009) study and ICA denoising of task fMRI data from the study by Drucker et al. (2019).

^2^
Based on recommendation from FSL experts (https://fsl.fmrib.ox.ac.uk/fsl/fslwiki/FIX/UserGuide)
