# Supplementary material for: A method to mitigate spatio‐temporally varying task‐correlated motion artifacts from overt‐speech fMRI paradigms in aphasia
Source: Hum Brain Mapp. 2020 Nov 19;42(4):1116–29. doi: 10.1002/hbm.25280 (PMC7856637; doi:10.1002/hbm.25280)
Supplement: Supplementary file 1 — Appendix S1. Supporting Information. [file HBM-42-1116-s001.docx]

**Supplementary Methods**

**S1.** Additional demographics and lesion heat map

Lesion size for each stroke participant is characterized in the table below. Automatic stroke lesion segmentations were generated using LINDA^1^ on the T1w images, followed by manual touch-up with itksnap^2^ to exclude healthy areas falsely identified as lesion and to include damaged regions falsely identified as non-lesioned. AFNI was utilized to calculate the lesion volume (= total number of voxels within the lesion map * voxel size; 3dmaskave), and to generate the lesion heat map for overlap across the 14 stroke subjects (3dOverlap).


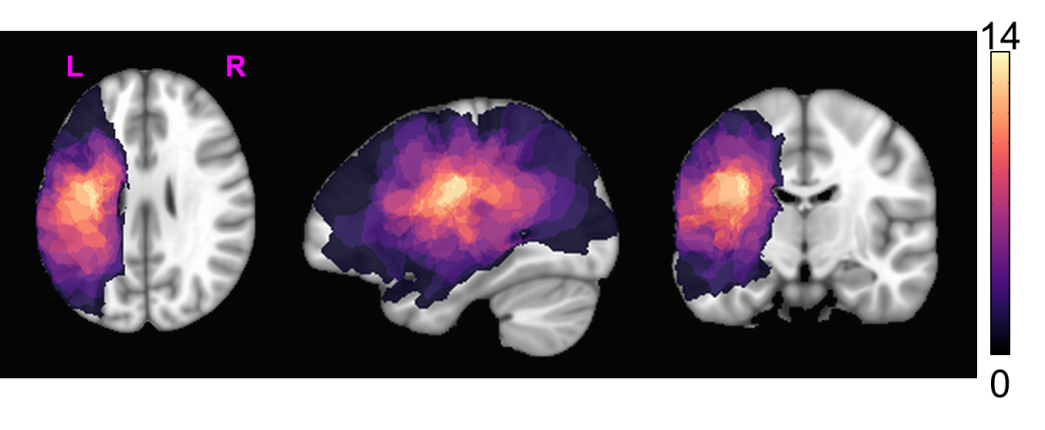


Figure S1: Depiction of lesion overlap across all 14 stroke subjects. The color bar indicates the overlap across all 14 participants.

| ID | Lesion vOLUME (C.C) |
| --- | --- |
| S01 | 25679.01 |
| S03 | 41787.92 |
| S04 | 151401.77 |
| S05 | 31856.34 |
| S06 | 74020.59 |
| S07 | 37448.53 |
| S08 | 172740.57 |
| S10 | 129734.51 |
| S11 | 98819.84 |
| S12 | 39737.89 |
| S14 | 19543.78 |
| S15 | 87975.48 |
| S16 | 57037.7 |
| S19 | 47619.44 |

**S2.** MELODIC setup for ICA decomposition of the task fMRI data

Multivariate Exploratory Linear Decomposition into Independent Components (MELODIC) is an FSL tool (Smith et al., 2004) that utilizes probabilistic ICA approach to decompose the 4D fMRI datasets into a set of spatial independent components (ICs) via z-statistical maps and associated time-courses. Within the MELODIC GUI, we employed the following major parameter setup for optimal results: (a) in the ‘Data’ tab, temporal high-pass filtering at 0.01Hz cut-off to remove contamination from low frequency drifts, (b) in the ‘Pre-Stats’ tab, the flag for motion correction (MCFLIRT) was turned on as we learned that this internal step was necessary for subsequent ICA classification using FSL FIX. We also employed smoothing (4mm kernel) to facilitate decomposition into signal and noise components. From a practical standpoint, smoothing also facilitates manageable number of components for hand classification. The BET based brain extraction flag was also turned on to obtain improved co-registration and edge detection, (c) in the ‘Registration’ tab, each of the functional run was spatially co-registered to the subject’s denoised and skull-stripped high resolution T1w image in native space using the boundary based registration algorithm. (d) in the ‘Stats’ tab, automatic estimation of the number of ICs (Beckmann and Smith 2004) was found to be optimal. The flag for variance normalization of time courses was turned off to maintain the task sensitivity, and (e) in the ‘Post-stats’ tab, we retained the default setting. Providing the design matrix with a ‘time-series model’ and ‘time-series contrast’ did not work optimally for this proposed algorithm, since the random runs chosen for hand classification and training had different stimulus timings.

**S3.** An approximated calculation of task frequency

As described in Methods section 2.4.2, calculation of task frequency is important in hand classification of task fMRI-based ICs. Although the inter-trial intervals (ITIs) were jittered, note that the length of each run was the same. Thus, an approximated calculation of task frequency (TF) is calculated as follows:

$$TF= \frac{1}{\frac{\left( \#of volumes \right)*\left( length of TR \right)}{\left( \#of trials \right)}}$$

In our study, each run comprised of 178 volumes, 10 trials and a TR of 1.7s. Plugging in these values into the above equation resulted with a task frequency (TF) = 0.03Hz.

**S4.** Detailed statistical output for HRF fitting

As described in methods sub-section 2.4.3, the estimated HRF from each methodology was fitted to a canonical HRF using non-linear curve fitting algorithm. Tabulated below is the detailed statistical output for the HRF fit parameters of Equation-1. Note that the parameter *A* was normalized to 1 to allow for fair comparison across methodologies and thus was not entered into the curve fitting algorithm. The partial and full-model p-values that were non-significant at an alpha of 0.05 are noted in red font color.

| **Methodology** | *α_1_* | | *α_2_* | | *β_1_* | | *β_2_* | | *c* | | Full model | |
| --- | --- | --- | --- | --- | --- | --- | --- | --- | --- | --- | --- | --- |
|  | p_partial_ | F_partial_ | p_partial_ | F_partial_ | p_partial_ | F_partial_ | p_partial_ | F_partial_ | p_partial_ | F_partial_ | p | F |
| TCMcorr | <0.000 | 28.42 | 0.04 | 4.11 | <0.000 | 22.97 | 0.04 | 4.4 | <0.000 | 60.69 | <0.000 | 33.95 |
| Standard | 0.009 | 6.84 | 0.2 | 1.7 | 0.01 | 6.55 | 0.15 | 2.03 | <0.000 | 50.82 | <0.000 | 22.94 |
| AROMA | <0.000 | 24.32 | 0.5 | 0.45 | <0.000 | 15.31 | 0.5 | 0.45 | <0.000 | 55.19 | <0.000 | 23.86 |
| TCMcorr+AROMA | <0.000 | 31.87 | 0 | 8.07E6 | <0.000 | 19.03 | 0 | 2.56E4 | <0.000 | 12.58 | 0 | 1.32E22 |

**S5.** Relationship between various clinical factors and TCM artifact and its removal

*WAB Fluency:*

The participant’s fluency, as measured outside of the scanner with WAB fluency, is significantly related to TCM artifact with a linear regression model in areas L-MFG (R^2^=0.43. p=0.01) and R-aSTG (R^2^=0.30. p=0.04). As is apparent in Figure S2, a greater TCM artifact is related to lower WAB fluency, indicating that aphasia related hesitations or paraphasias may be encoded with a greater TCM artifact. Other sampled brain areas do not encode such a relationship in this cohort, including L-PMd, L-SFG, mPFC, and R-PTr.

**
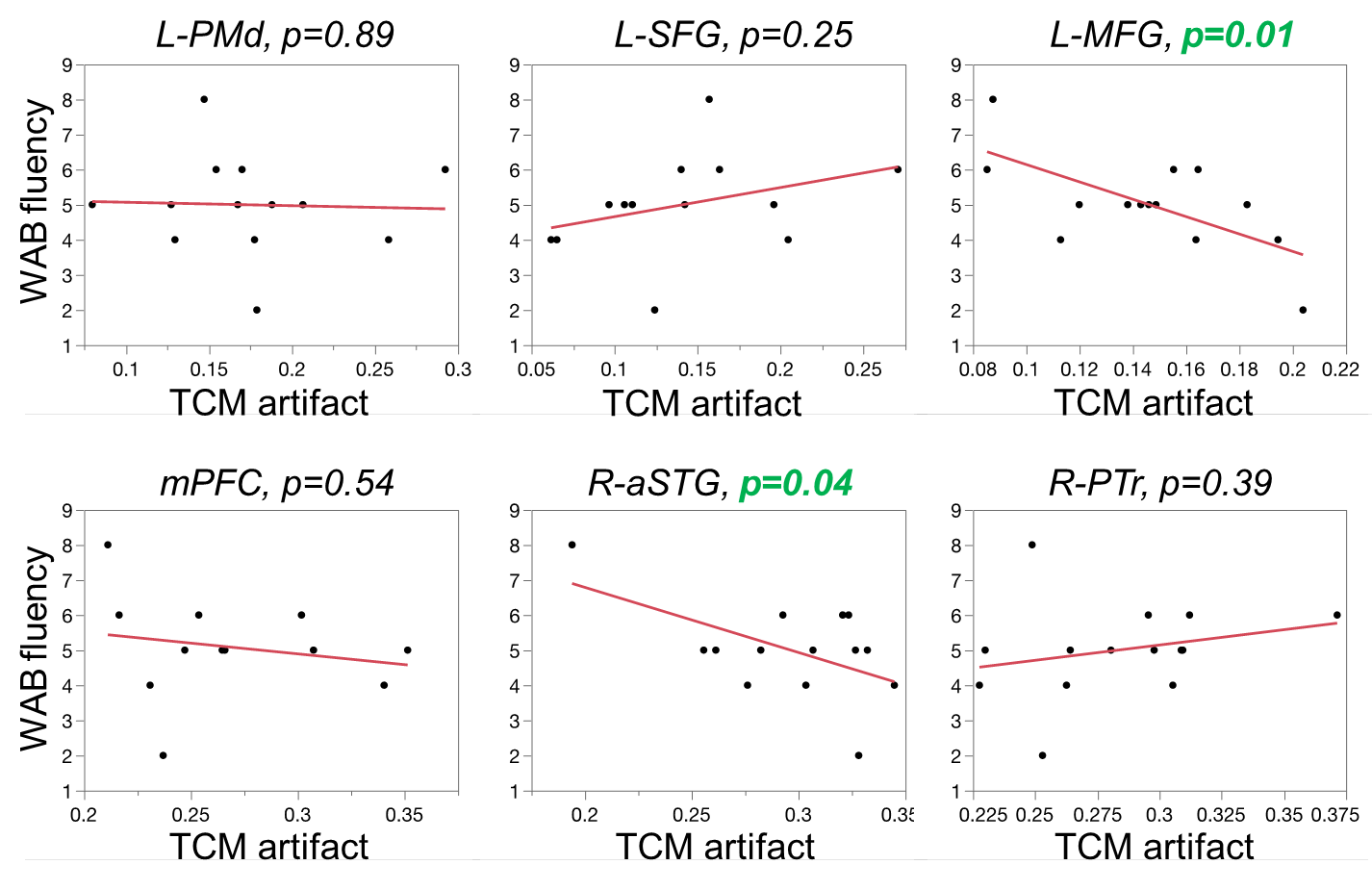
**

Figure S2 : Linear relationship between WAB fluency and TCM artifact for 6 sampled brain regions. Note: L-PMd=left dorsal pre-motor, L-SFG=left superior frontal gyrus, L-MFG=left middle frontal gyrus, mPFC=medial prefrontal cortex, R-aSTG=right anterior superior temporal gyrus, R-PTr=right pars triangularis. The TCM artifact is the Fano Factor derived from Standard approach.

The two areas that showed a significant relationship were promoted to determine if removal of TCM artifact affected the relationship. The table below shows the effects of Methodology on the relationship between denoised signal and WAB fluency.

| **Methodology** | *L-MFG* | | *R-aSTG* | |
| --- | --- | --- | --- | --- |
|  | R^2^ | p | R^2^ | p |
| TCMcorr | 0.31 | 0.04 | 0.23 | 0.08 |
| AROMA | 0.39 | 0.02 | <0.01 | 0.93 |
| TCMcorr+AROMA | .41 | 0.01 | 0.02 | 0.66 |

*Lesion Size and WAB AQ:*

The whole model for TCM artifact and its relationship to aphasia severity (WAB AQ) and lesion size was not significant, although L-MFG (F(2,11)=2.08, p=0.17) and R-aSTG (F(2,11)=2.19, p=0.16) were trending. Subsequent t-tests revealed a trending effect for aphasia severity in L-MFG (t(13)=-1.94, p=0.08) and R-aSTG (t(13)=-2.08, p=0.06). Lesion size was not significantly related to TCM artifact with this model. The variance inflation factor for each variable was below 3 and therefore does not warrant corrective measures. A graphical summary of the effects can be seen in Figure S3.


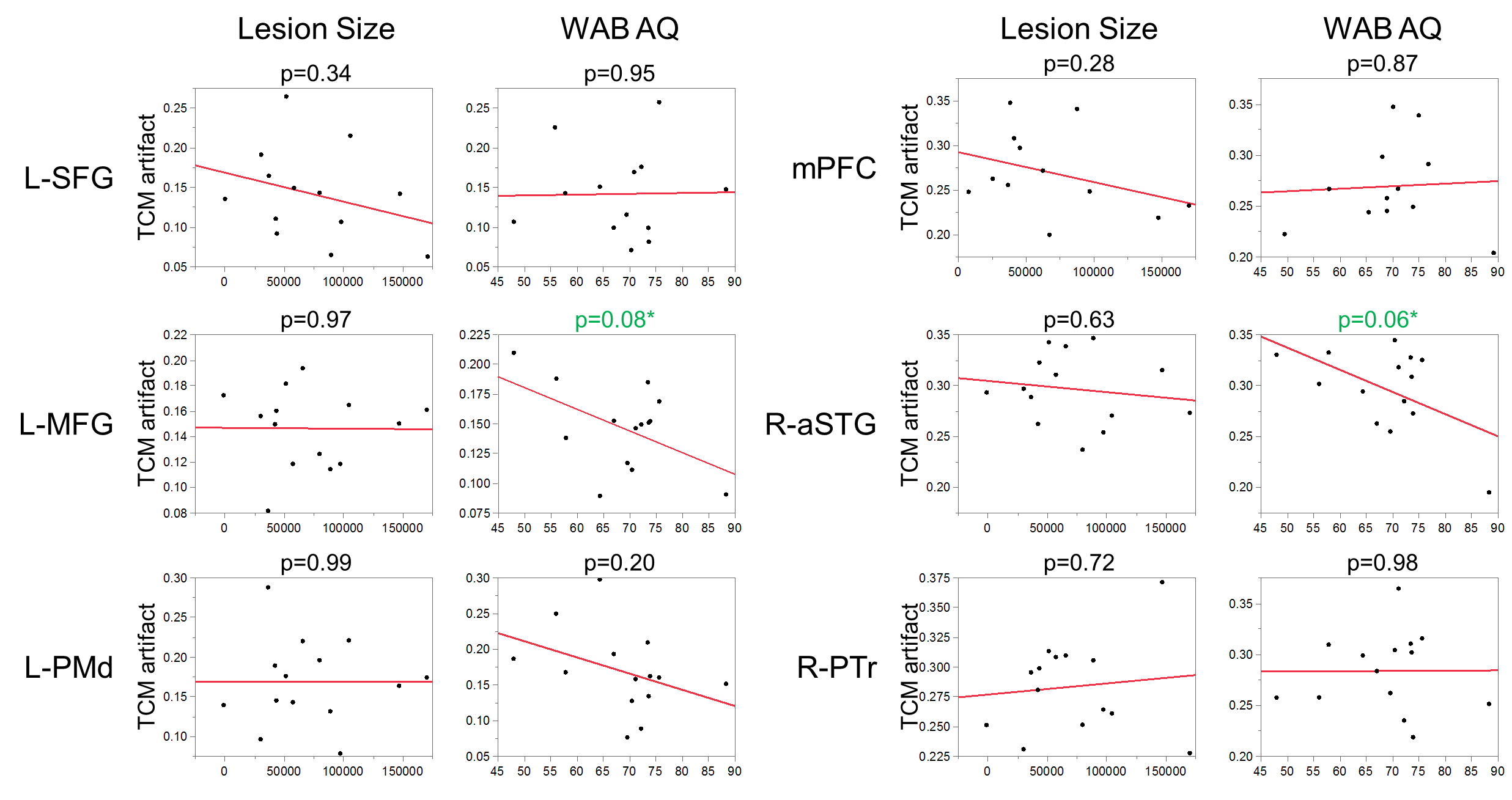


Figure S3 : Leverage effect plots of TCM artifact relationship with Lesion size and aphasia severity (WAB AQ). * denotes tending at p of 0.05. The TCM artifact is the Fano Factor derived from Standard approach.

The two areas that showed a trending relationship were promoted to determine if removal of TCM artifact affected the relationship. The table below shows the effects of Methodology on the relationship between WAB AQ, lesion size, and signal.

| **Methodology** | *L-MFG* | | | | | | *R-aSTG* | | | | | |
| --- | --- | --- | --- | --- | --- | --- | --- | --- | --- | --- | --- | --- |
|  | *Full model* | | *Lesion size* | | *WAB AQ* | | *Full model* | | *Lesion size* | | *WAB AQ* | |
|  | F | p | t | p | t | p | F | p | t | p | t | p |
| TCMcorr | 3.61 | 0.06 | 1.39 | 0.19 | -1.73 | 0.11 | 0.91 | 0.43 | -0.45 | 0.66 | -1.35 | 0.20 |
| AROMA | 6.72 | 0.01 | -2.06 | 0.06 | -3.53 | 0.005 | 0.69 | 0.52 | -0.95 | 0.36 | -0.95 | 0.36 |
| TCMcorr+AROMA | 4.94 | 0.03 | -1.07 | 0.30 | -3.14 | 0.01 | 0.29 | 0.75 | -0.46 | 0.66 | -0.73 | 0.48 |

**S6.** Relative thresholding and task activation

The relative task activation threshold (R^2^) was decreased in a stepwise fashion between 0.16 to 0.12 on the proposed TCMcorr denoising approach, with cluster size 30 for all the R^2^s. The sagittal montage view covering the lesion and language eloquent areas are shown below. Note: The orange colored label in each figure denotes the subject ID.


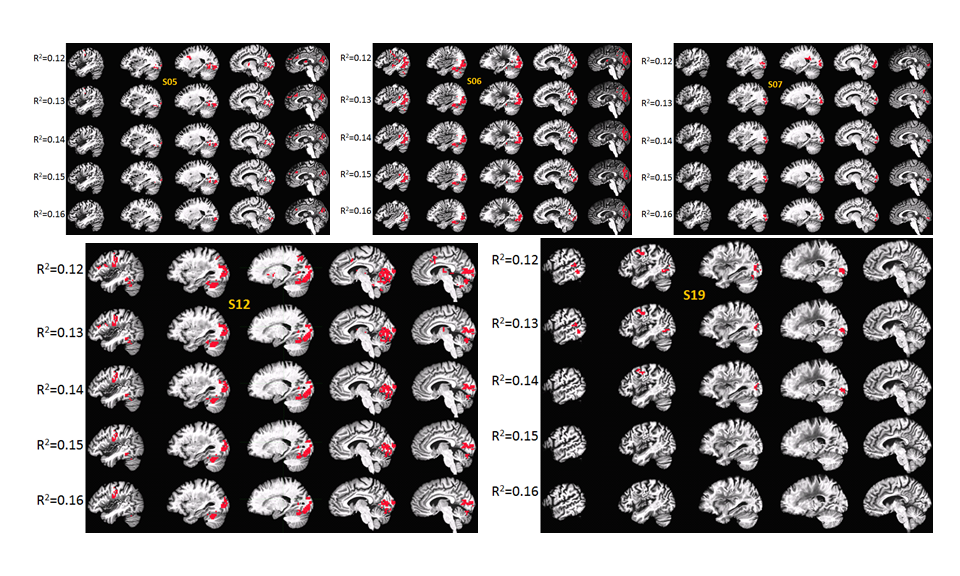


**S7.** Task activation maps across all 14 subjects

Individual subject level task activation maps (R^2^ = 0.16 and cluster size=30) across all 4 methodologies are shown below. The axial montage view shows both the lesion and language eloquent areas. Note: The orange colored label in each figure denotes the subject ID.


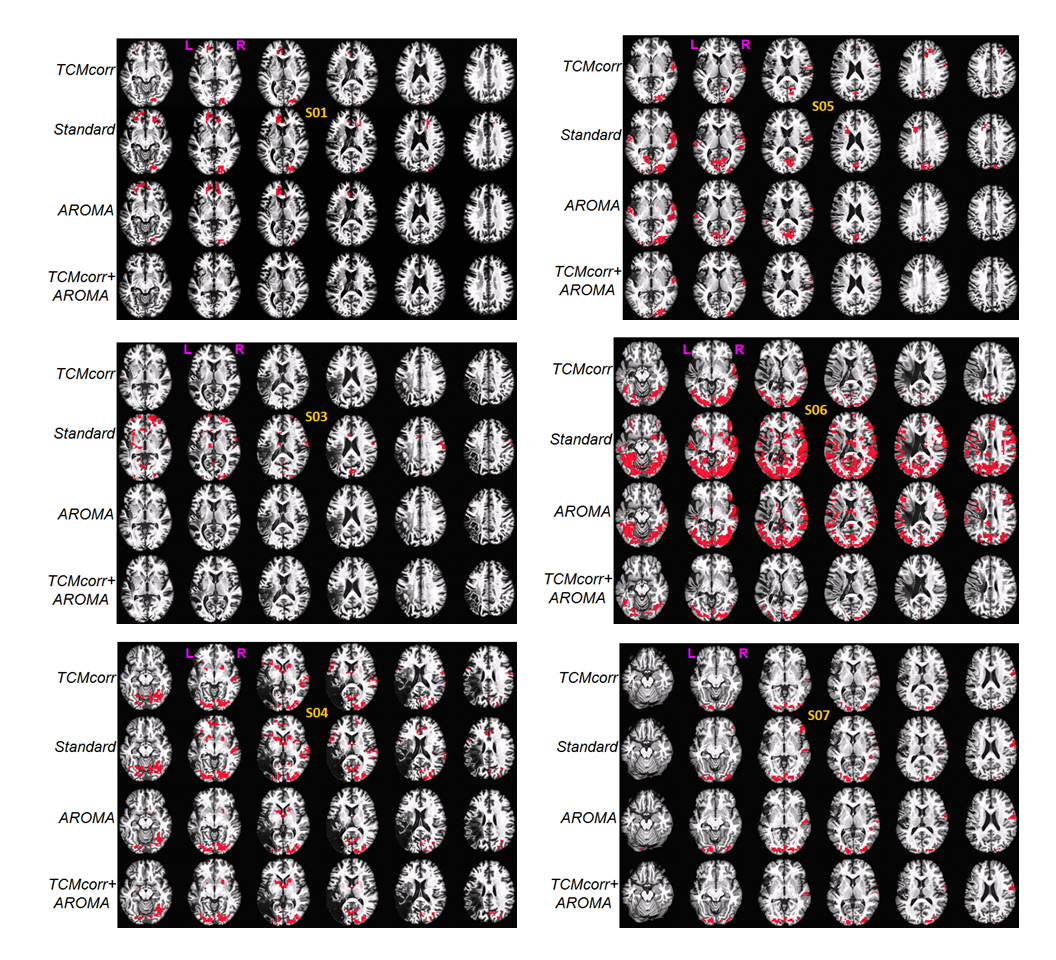


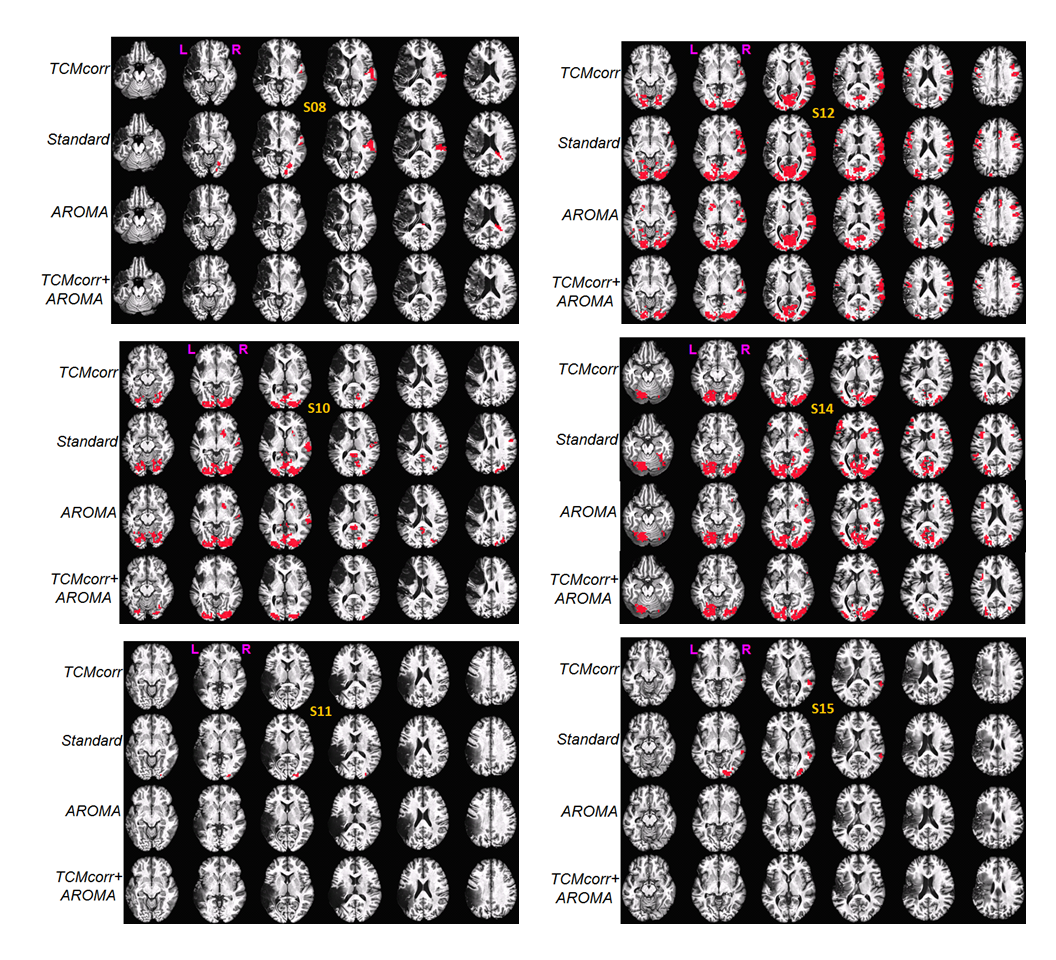


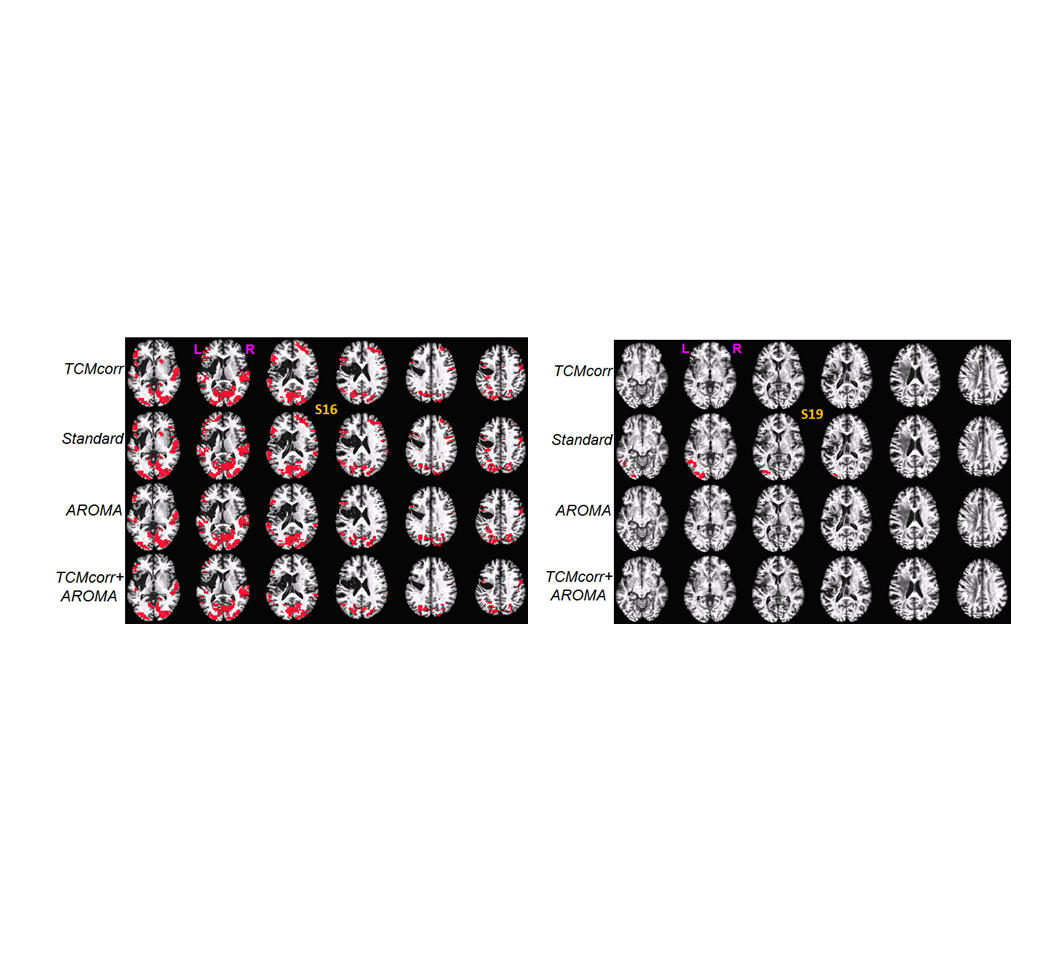


**References**

1. Pustina D, Coslett HB, Turkeltaub PE, Tustison N, Schwartz MF, Avants B. Automated segmentation of chronic stroke lesions using LINDA: Lesion identification with neighborhood data analysis. *Hum Brain Mapp.* 2016;37(4):1405-1421.

2. Yushkevich PA, Piven J, Hazlett HC, et al. User-guided 3D active contour segmentation of anatomical structures: significantly improved efficiency and reliability. *Neuroimage.* 2006;31(3):1116-1128.
